# Hepatic vasculitis mimicking liver abscesses in a patient with systemic lupus erythematosus

**DOI:** 10.4103/0256-4947.57172

**Published:** 2009

**Authors:** Thari Alanazi, Mohammad Alqahtani, Huda Al Duraihim, Khalid Al Khathlan, Bader Al Ahmari, Dorothy Makanjuola, Mohammad Afzal

**Affiliations:** a From the Division of Internal Medicine, King Abdulaziz Medical City, Riyadh, Saudi Arabia; b From the Division of Rheumatology, Department of Medicine, King Abdulaziz Medical City, Riyadh, Saudi Arabia; c From the Department of Radiology, King Abdulaziz Medical City, Riyadh, Saudi Arabia; d From the Department of Pathology, King Abdulaziz Medical City, Riyadh, Saudi Arabia

## Abstract

Clinical and radiological liver diseases are uncommon in patients with systemic lupus erythematosus (SLE). We report a 29-year-old female with SLE who presented with right upper quadrant abdominal pain, thrombocytopenia, elevated liver enzymes and multiple hypodense lesions in the liver on a computed tomography (CT) study that mimicked multiple liver abscesses. A liver biopsy showed mild chronic inflammation. Culture results were negative. With steroid therapy the patient improved clinically, the platelet count returned to the normal range and the multiple liver lesions disappeared radiologicaly. This patient represents a rare case of SLE that had hepatic vasculitis mimicking multiple liver abscesses.

Systemic lupus erythematosus (SLE) is a chronic inflammatory disease of unknown cause which can affect the skin, joints, kidneys, lungs, nervous system and other organs of the body. Immunologic abnormalities, especially the production of a number of antinuclear antibodies, are another prominent feature of the disease. The clinical course of SLE is variable and may be characterized by periods of remissions, chronicity or acute relapses. Women, especially in their 20s and 30s, are affected more frequently than men. Although biochemical evidence of liver disease is common in patients with SLE, clinical liver disease is uncommon.^1^ We reported here a case of SLE that presented with right upper quadrant pain and multiple liver lesions secondary to hepatic vasculitis.

## CASE

A 29-year-old woman known to have SLE for 6 years presented to the emergency department with a 5-day history of upper abdominal pain. She described the abdominal pain as dull and crampy, mild to moderate in intensity and localized to the epigastric and right upper quadrant regions. Two days before admission, the abdominal pain became constant and severe, and associated with nausea and vomiting but not associated with positional changes, eating, bowel movements or other factors. There had been no fever, chills, diarrhea, heartburn, hematemesis, melena or hematochezia. There was no history of herbal use, taking any new drugs or ingestion of unusual or undercooked foods and no recent travel or contact with ill persons. She was not known to have drug allergies and had no history of cigarette use, alcohol drinking or drug abuse.

She had been discharged from the hospital 2 weeks previously due the intrauterine death of her fetus at 30 weeks gestational age. She had been treated for SLE with low-dose prednisolone, with the addition of azathioprine and hydroxychloroquine and was maintained on pantaprazole as gastric prophylaxis. Her family history was notable for her mother who also had SLE.

On physical examination, the patient was afebrile. Her blood pressure was 158/90 mm Hg, pulse rate was 117 beats per minute, respiratory rate was 22 breaths per minute and oxygen saturation was 97% while breathing room air. The patient appeared to be in severe pain, with a pain score 8/10, and there were no jaundice, pallor, skin rash or mouth ulcers. The lungs and heart sounds were normal. Abdominal examination yielded right upper quadrant tenderness with no guarding or rebound tenderness, normal bowel sounds and no palpable organomegaly or masses. Results of the remainder of the complete multisystem examination were unremarkable.

**Figure 1:**
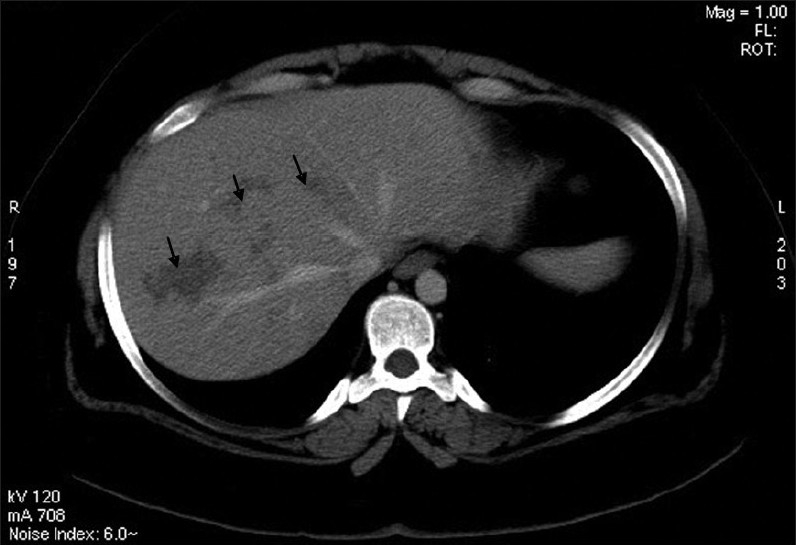
A computed tomography scan of the abdomen with intravenous contrast presents hypodensities with no appreciable enhancement distributed in an irregular patchy and linear form. Some of the distribution simulates the cluster sign of abscesses.

Laboratory studies revealed the following: serum sodium 132 mmol/L, potassium 4 mmol/L, and creatinine 54 μmol/L. A complete blood cell count revealed a hemoglobin level of 103 g/L, mean corpuscular volume 90 fL, mean corpuscular hemoglobin 30 pg, a white blood cell count of 10.8×10^9^/L, an absolute neutrophil count of 9×10^9^/L, lymphocyte count of 1×10^9^/L, and an eosionophil count of 0.2×10^9^/L, and a platelet count of 39×10^9^/L. The hemolysis markers were normal. Serum iron saturation was 30%. Prothrombin time was 9.9 sec, partial thromboplastin time 34.2 sec, international normalized ratio of 1.1 and the fibrinogen level was normal. Serum liver biochemistry results showed a total bilirubin of 23.6 μmol/L and a direct bilirubin of 13.1μmol/L, alkaline phosphatase of 161 U/L, aspartate aminotransferase of 354 U/L, alanine aminotransferase of 429 U/L, albumin of 37 G/L. Serum amylase was normal. Stool examination for ova, parasites, and occult blood were negative. Urine analysis was normal. The erythrocyte sedimentation rate was elevated 125 mm/h. The complement levels were normal. Serologic studies revealed a positive antinuclear antibody test of 123.7 units and anti-ds-DNA test 200 units. Anti-SM, anticardiolipin antibody, antimitochondrial antibody, and anti-smooth muscle antibody were all negative. Immunoglobulin G was elevated at 26.5 g/L, and immunoglobulin M was normal. Viral hepatitis B and C screening tests were negative. The thyroid function test, vitamin B12 and folate levels were all normal. A chest radiograph, electrocardiogram and serum cardiac enzymes were all within normal limits. A computed tomography scan of the abdomen showed a normal liver size with multiple irregular hypodense lesions, mainly in the right hepatic lobe, which did not show arterial or venous enhancement (Figure 1)

The patient was diagnosed initially with SLE flare up with liver involvement and autoimmune thrombocytopenia. She was started on high-dose methylprednisolone, 1 gram intravenously daily for 5 days, and intravenous immunoglobulin for 3 days. On the second day of management the pain dramaticaly disappeared and the liver enzymes started to improve as well as the platelet count. The patient initially was covered with broad spectrum intravenous antibiotics (pipracillin-tazobactam and metronidazole) due to the possibility of pyogenic or amoebic liver abscess, but since there was no evidence of infection, clinically or by laboratory investigation, and the her condition improved clinically and biochemically in the second day after receiving pulse steroids, the antibiotics were discontinued.

An ultrasound-guided biopsy from the liver lesions was non-conclusive, showing mild congestion and chronic mild inflammation with no granulomatous or atypical changes. Results of cultures from blood, urine and stool were negative. Amebiasis antibody was negative. She was discharged in good condition with no pain and normal liver enzymes and platelet count. Discharge medications included prednisolone tablet 40 mg daily and resumption of previous medications. A follow up CT-scan abdomen one month from discharge showed complete resolution of the previous lesions in the liver with normal liver parenchyma.

## DISCUSSION

Although overt liver disease has been rarely described in patients with SLE, subclinical manifestations or biochemical abnormalities are not uncommon. In one retrospective study of 238 SLE patients, 60% were found to have liver function abnormalities, with 24% having presenting symptoms consistent with hepatitis. The diagnosis of liver disease in SLE usually occurred one or more years after the diagnosis of SLE.^1^ The presence of elevated liver enzymes and clinical symptoms of hepatitis correlated with a lupus flare in 21%.^1^ In another retrospective study of 81 cases, liver abnormality might have been related to SLE itself in 19 of 45 cases who had abnormal liver enzymes.^2^ In a prospective study, hepatic enzyme elevation was observed in 23% of 260 lupus patients, of which the cause of liver enzyme abnormalities was not identifiable in 8%. However, this cause was related to the activity of lupus in 8% of the cases,^3^ and the majority of biochemical abnormalities seen in these studies were usually secondary to other causes such as drug-induced liver dysfunction, alcohol, liver congestion from congestive heart failure, infection or metabolic disturbances. Hepatomegaly (39% to 42%) and jaundice (24%) were often seen at the onset of liver disease.^1^,^2^ Various forms of hepatic pathology related to SLE have been described, and they include fatty liver,^2^,^4^ portal inflammation,^2^ chronic active hepatitis,^1^,^2^ chronic persistent hepatitis,^1^,^4^ cirrhosis,^1^ cholestasis,^1^,^4^ hepatic necrosis associated with antiphospholipid antibody syndrome,^5^ granulomatous hepatitis,^1^ non-specific reactive hepatitis,^4^ nodular regenerative hyperplasia,^4^ hepatic infarction^4^ and arteritis.^4^,^6^,^7^

Although granulomatous hepatitis has been described in SLE, it is uncommon. It was found in 4 of 33 (12%) histological-proved liver diseases in SLE reported by Runyon et al.^1^ Two of these patients also had hepatomegaly, and one had a lupus flare. Feurle et al reported a case of granulomatous hepatitis, which manifested biochemically as an elevation of alkaline phosphatase during a flare-up of SLE.^8^ Among the older reports, Harvey et al found two cases of hepatic vasculitis and two of hepatic granulomas.^7^ Kofman et al described granulomatous hepatitis in one of 11 liver pathological specimens.^9^ In Aronson's series of 19 autopsy-proved cases of SLE, hepatic granuloma was observed in 5%. None of these cases had severe liver diseases.^10^

Conflicting data on the incidence of hepatic vasculitis in SLE have been reported. In a report of the 33 histologically proved liver diseases in SLE by Runyon et al, none had vasculitis.^1^ In contrast, hepatic arteritis was found in 11 cases from 52 (21%) pathologically proved liver diseases in SLE from the Japanese autopsy registry.^4^ Miyake et al reported a young Japanese man with acute SLE, elevated transaminase, and multiple hypodense spots in the liver in a CT study.^11^ A biopsy specimen from a low-density spot lesion showed mild inflammatory cell infiltration and piecemeal necrosis.These hypodense lesions may be due to vasculitis, as corticosteroid therapy improved the patient's clinical status, normalized laboratory abnormalities and caused the liver lesions to disappear. Fehr et al reported a case of liver necrosis in an SLE patient with antiphospholipid syndrome, who also had HELLP (Hemolysis, Elevated Liver enzymes and Low Platelet count)-like syndrome. A CT scan of the liver also showed multiple hypodense lesions, and biopsy revealed periportal necrosis with fibrin deposit and accumulation of sinusoidal granulocytes. Anticoagulant and immunosuppressive therapy improved the patient's clinical status.^5^ The hypodense lesion in this case might be related to hepatic infarction secondary to antiphospholipid syndrome. Spontaneous rupture of the liver, secondary to hepatic vasculitis, has been reported by Levitin et al.^6^ However, the imaging study of the liver was not performed in this case.

Suparaporn et al reported a patient with SLE who had hepatic vasculitis with necrosis mimicking multiple liver abscesses as an initial presentation. Because of suspicion of pyogenic liver abscess the patient was not given steroid therapy and unfortunately she died. A biopsy specimen showed necrotizing granuloma with a large area of necrosis.^12^ In our case the main concern was starting the patient on a high dose of corticosteroid because the pyogenic liver abscess was part of the differential diagnosis. However, because of the clinical scenario and the presence of autoimmune thrombocytopenia, high anti-DNA (Deoxyribonucleic acid), which indicates active SLE, and negative septic work up, the decision was to give the patient pulse steroid and IV immunoglobin. In addition to that the patient was initially covered with intravenous antibiotics for three days, which were discontinued because of the dramatic response to pulse steroids in a very short time. The lack of significant enhancement of the lesion is supportive of the absence of an infectious component and the linear fashion suggests possible periportal vascular insult. The negative test for anticardiolipin antibody and lupus anticoagulant made the possibility of hepatic infarction secondary to antiphospholipid syndrome unlikely.

The patient was discharged on high dose of oral steroid for one month. The repeated CT scan four weeks later showed complete resolution of the these multiple liver lesions. The presence of active SLE disease and the complete recovery (clinically and radiologicaly) with high-dose steroid supported the diagnosis of hepatic vasculitis as a cause of this patient's multiple liver lesions, which mimic multiple liver abscesses.
